# Assessment of Medical Professionalism: Development and psychometric analysis of Professionalism Assessment Tool (PAT) in Pakistani context using Delphi Techniques

**DOI:** 10.12669/pjms.39.2.6608

**Published:** 2023

**Authors:** Khola Noreen, Rukhsana W Zuberi, Kausar Aftab, Sabahat Fatima

**Affiliations:** 1 Dr. Khola Noreen, Associate Professor, Community Medicine, Rawalpindi Medical University, Rawalpindi, Pakistan; 2 Prof. Dr. Rukhsana W Zuberi, Professor Emerita, Aga Khan University, Karachi, Pakistan; 3 Dr. Kausar Aftab, Assistant Professor, Community Medicine, Gujranwala Medical College, Gujranwala, Pakistan; 4 Dr. Sabahat Fatima, Assistant Professor, Biochemistry, Gujranwala Medical College, Gujranwala, Pakistan

**Keywords:** Content validity, Reliability, Assessment, Professionalism, Undergraduate, Medical student

## Abstract

**Objective:**

This study aims to develop and assess the content validity along with the reliability of a Professionalism Assessment Tool (PAT) with an intention to measure professionalism among undergraduate medical students.

**Methods:**

This psychometric analytical study validation study was carried out at Rawalpindi Medical University from 1^st^ February to 1^st^ June, 2021 after establishing feasibility and obtaining ethical approval. The non-probability convince sampling was employed to collect data. Using Nunnally’s, the ratio of e subjects per item was selected, as our preliminary tool has 48 items so 384 sample size was estimated for scale validation. The preliminary 48-item tool with five subscales(cSS) developed through mutual consensus by the Delphi technique namely Communication skill(cSS1),-7 item, Accountability(cSS2)-8 item, Altruism(cSS3)-13 item, Self-Directed Learning (cSS4)-10 item and Ethics(cSS5),-10 item was labelled as Professionalism Assessment Tool (PAT). The tool was administered to 4^th^ year MBBS students, the data obtained was analyzed by calculating Cronbach’s alpha to estimate the reliability. The SPSS version 26 was used for data analysis.

**Results:**

The 48-item PAT had an overall reliability (Cronbach’s alpha) of 0.783. The Reliability of the new Subscales were communication skills (0.405), self-directed learning (0.527), Accountability (0.378), Altruism (0.486) and Ethics (0.715).

**Conclusion:**

The final tool developed for assessment of professionalism had 48 items on a seven point Likert like scale, across five Subscales. Results showed that it was determined as a useful tool in assessing professionalism in undergraduate medical students to generate reliable results for valid decision-making.

## INTRODUCTION

The last decade has seen a significant shift in the way the medical profession and professionalism is defined and practiced. These shifting trends reflect a change in the way health-care is delivered world-wide, as well as the changed expectations of the public and community from the health-care professionals.^1^

The accreditation councils, regulatory and licensing bodies and medical associations around the globe have emphasized the incorporation of professionalism in medical curriculum.^2^ The importance of the impact of sociocultural differences on the definition of professionalism and its assessment has been discussed and debated at various platforms but there is real dearth of literature with regards to assessment of professionalism in the multi-ethnic Asian context.^3^ A study from Singapore evaluated professionalism using already validated tool namely Professionalism Mini Evaluation (P-MEX) and reported that most but not all the items of P-MEX were found relevant in their assessment of professionalism, and highlighted the need to include the empathy and collegiality while assessing professionalism.^4^ When P-MEX was validated in Japan, it was modified and new items were added. The new items helped to achieve adequate validity of the factors.^5^

Professionalism has been included as a core competency framework in both the Pakistan Medical Commission (PMC)^6^ and College of Physician and Surgeons (CPSP)^7^ curricula, yet these frameworks are not elaborated enough to define the constituent domains and sub domains of professionalism. Professionalism is closely related to the social contract, and Pakistani medical fraternity may perceive professionalism differently from others.^8^ Since no study has been conducted to identify the components of professionalism in the context of medical education in Pakistan, recent study conducted by Butt proposed revalidation of the ‘Arabian Learners Attitude on Medical Professionalism Scale (LAMPS), which after validation in Pakistan, may be called Pak-LAMPS.^9^ As professionalism is influenced by the social context, the social context of Saudi Arabia and Pakistan are vastly different, so it cannot used in Pakistani Context^8^. Although PMEX has been widely used and has been revalidated in various countries but it seems that P-MEX may be useful in the western culture, but may not completely fit the eastern context, and warrants the development of different tool for assessment of professionalism in sociocultural context of Pakistan.^3^,^10^

This study aimed to develop and validate a tool for assessment of professionalism in undergraduate medical students. To the best of our knowledge, this is the first study which attempts to operationalize a conceptual framework which would illustrate the components of professionalism in the Pakistani context.

## METHODS

This psychometric analytical study was carried out at Rawalpindi Medical University (RMU) after establishing feasibility and obtaining ethical approval from both RMU (244/IREF/RMU/2020) and Aga Khan University (AKU), (2021-5690-16626). Data was collected from February 1^st^, 2021 to June 1^st^, 2021. This study focuses on the result of initial pilot, which was run on year four MBBS medical students, while completing the Community Medicine rotation, so consecutive non-probability sampling was employed to collect data. Using Nunnally’s,^11^ the ratio of eight subjects per item was selected, as our preliminary tool has 48 items so 48x8=384 sample size was estimated for scale validation. After taking informed written consent, the tool was administered to the undergraduate medical (Year IV) students at the end of four Week Research Project Rotation. Each research batch comprised of thirty students who work under direct supervision of assigned supervisor during this period. The Cronbach’s alpha was used to calculate the reliability of the results obtained by using SPSS. Different cut offs are used depending upon nature of examination. 0.90 for very high stakes exams, 0.80–0.89 is acceptable for moderate tests, 0.70–0.79 would be acceptable for lower stakes tests. Validity (content) was established during Delphi rounds.

### Steps in Tool development:

### 1. Selection of project (Development of Professionalism Assessment Tool (PAT):

The tool refers to a “data collection instrument comprising of predetermined set of questions that is used for collection and record of information about particular issue of concern”.^12^ There are number of tools available in literature for assessment of professionalism but there is real dearth of literature with regards to availability & practicality of such tool in our part of world.

### 2. Planning and blueprinting:

This process involves clearly mentioning the inclusion/exclusion criteria of checklist, exploring literature to gain knowledge of various domains of professionalism to construct themes and subthemes, initial plan for scoring rubrics and rating scales

### 3. Tool development:

Tool development involved following sequential order:

### Extensive literature review

In order to identify potential items to be included in tool, comprehensive review of current literature and theories was conducted. The questionnaire was originally developed by extensive literature review of already available tools for assessment of professionalism during contact session of assessment course of MHPE via consultative process involving expert opinion from group members. The tool was modified and revised on the basis of feedback from the faculty of diverse specialties and medical educationists. Initially, sixty items were found and listed after extensive literature review.

### Reconcile & synthesize the literature review

Reconciling and synthesizing of the literature was done and items were synchronized into conceptual framework and grouped into domains and sub domains to develop the tool.

### Devising an items

After selecting five themes of professionalism, item was generated to represent the construct of each domain. Term “item” refers to questions that are pertinent to each domain of professionalism. Literature has cited number of guidelines for writing items.^12^ For each domain more items were developed (e.g. developing 15 potential items with the hope that ultimately nine or ten left) based on expert opinion of facilitators and feedback from other group members.

### Selection of response scale

Evidence has supported that performance based assessment can be evaluated by checklist alone or in combination with Global rating scale.^12^ Checklist tend to reduce examiner subjectivity; hence checklist was used as Likert-type scale in which responses are anchored according to degree of agreement or frequency of an event. Seven-point Likert type scale was used to record the responses with the legends of: ‘not observed, seldom, sometimes, usually, very frequently, almost always, always.’ Global / holistic rating was introduced as an evaluative tool for an overall impression of the faculty member regarding the student, thus further validation of the tool.

### Delphi Method for Content Validation:

Content validity was established by both quantitative and qualitative method. In Quantitative method, the Content validity ratio is measured for selection of the most appropriate content. The formula of Content Validity Ratio CVR= (N_e_ - N/2)/ (N/2), *where N_e_* is the number of panellists indicating “essential” and N is the total number of panellists was used.^12^ CVR ratio also varies with the number of experts assessing the content. For a five-member expert panel, the minimum CVR required is 0.99. For a fifteen members expert panel, the minimum CVR required is 0.49, and for a 40 member expert panel, the minimum CVR required is 0.29. In Qualitative method, experts of the area review the content and give their expert opinion (subjective judgment) whether the tool measures what it is supposed to measure and whether the tool appears to measure what is should be measuring (face validity). Three rounds of Delphi technique were conducted to reach a consensus regarding the importance of the various items. All rounds were conducted by sharing the sixty items related to professionalism with fifteen faculty members of the department who were subject specialists with expertise in research.

### First round of Delphi:

In first round experts were requested to review the tool, during this process they mark all those items which according to their opinion should be removed from tool, items which are repetition among the subscales, those need editing or rephrasing were also highlighted, items which need exclusion were also marked. While devising items, it was specially kept in consideration that items should be short, simple, precise and written in language accustomed to the most of the target respondents. “Double-barrelled” items were avoided. Items assessing more than one domain simultaneously were removed. Leading questions that can result in biased responses were avoided. Homogenous items in which all participants respond similarly were removed as the small variance generated which will provide limited information about construct being assessed.^13^

### Second round of Delphi:

Qualified and experts review of the initial pool of items was carried out for further refinement. Content validity ratio is measured for selection of the most appropriate content. The formula of content validity ratio CVR= (N_e_ - N/2)/ (N/2) was used, responses were analyzed on Likert scale with each rating point was allocated with a particular score: 4=very important, 3=important, 2=somewhat important, 1=unimportant for clarity and necessity. An open ended question encouraging panelists to suggest additional components was included at the end of the questionnaire. Items were reviewed and revised to make sure about clarity of content, item construction and grammatical correctness. The purpose of expert review is to remove grammatical errors, biased and unclear items. The expert then develops mutual agreement on items included to enhance face and content validity. After mutual consensus of experts, the tool is reviewed, revised and after discussion, consensus was made on final version of questionnaire accordingly. There were 15 expert panelists, so items with CVR greater than 0.49, were accepted.^12^

### Third round of Delphi:

The third round Delphi established consensus on the final version of the tool for assessment of professionalism in which 48 of the total 60 items were retained.

In addition, five subscales were identified by consensus labelled as Consensus Sub Scale (cSS): cSS1: Communication skills, cSS2: Self-Directed Learning, cSS3: Accountability, cSS4: Altruism, cSS5: Ethics.

### Pilot Testing:

A pilot test of tool was done on small subset of (30-50) participants to remove unclear, ambiguous items and to review and revise improve the tool further. Improvement was done on basis of feedback of the respondents. Feasibility issues were also addressed, presence of floor (all responses scored at bottom) or ceiling effects (all scores aggregated at top) are also reviewed during pilot test to enhance feasibility and content validity.

## RESULTS

The study included 345 participants with overall mean age of 23.2 ± 2.3 years. Majority of the study participants were females 226 (65.5%) and males were 119 (34.5%). Pilot test was run on the students of year four MBBS.

### Calculation of Content Validity Ratio (CVR) of 60-itemed preliminary Tool:

The Content validity ratio is measured for selection of the most appropriate content. The formula of content validity ratio CVR= (N_e_ - N/2)/ (N/2), *where N_e_* is the number of panellists indicating “essential” and N is the total number of panellists was used. CVR ratio also varies with the number of experts assessing the content. Since there were fifteen-member expert panel, the minimum CVR required is 0.49, and for a 40-member expert panel, the minimum CVR required is 0.29.

### Descriptive statistics and Cronbach’s alpha of subscales of PAT consisting of 48 items (n= 345):

Mean, standard deviation, number of items and Cronbach’s alpha of each domain of the PAT is shown in Table-I. Reliability Analysis of the 48-Item Pilot Tool and its five subscales was conducted. The Cronbach’s alpha was used to calculate the reliability of the results obtained. Cronbach’s alpha of the 48-item Pilot Tool was 0.782, and Cronbach’s alphas of the subscales-by-consensus (cSS) are given in Table-II.

**Table-I:** Calculation of Content Validity Ratio (CVR) of 60-itemed preliminary Tool.

Item	Rating 3 or 4 Very important/ important	Rating 1 or 2 Somewhat important/not important	CVR = (Ne-/2)/(N/2)*	Interpretation
1.	12	3	0.8	Appropriate
2.	14	1	0.93	Appropriate
3.	15	0	1	Appropriate
4.	8	7	0.53	Appropriate
5.	12	3	0.8	Appropriate
6.	11	4	0.73	Appropriate
7.	8	7	0.53	Appropriate
8.	8	7	0.53	Appropriate
9.	11	4	0.73	Appropriate
10.	8	7	0.53	Appropriate
11.	13	2	0.86	Appropriate
12.	8	7	0.53	Appropriate
13.	9	6	0.6	Appropriate
14.	5	10	0.33	Eliminated
15.	11	4	0.73	Appropriate
16.	13	2	0.86	Appropriate
17.	10	5	0.66	Appropriate
18.	8	7	0.53	Appropriate
19.	14	1	0.93	Appropriate
20.	5	10	0.33	Eliminated
21.	13	2	0.86	Appropriate
22.	14	1	0.93	Appropriate
23.	8	7	0.53	Appropriate
24.	3	12	0.2	Eliminated
25.	14	1	0.93	Appropriate
26.	8	7	0.53	Appropriate
27.	13	2	0.86	Appropriate
28.	15	0	1	Appropriate
29.	14	1	0.93	Appropriate
30.	8	7	0.53	Appropriate
31.	2	13	0.13	Eliminated
32.	12	3	0.8	Appropriate
33.	11	4	0.73	Appropriate
34.	9	6	0.6	Appropriate
35.	15	0	1	Appropriate
36.	14	1	0.93	Appropriate
37.	11	4	0.73	Appropriate
38.	2	13	0.13	Eliminated
39.	14	1	0.93	Appropriate
40.	4	11	0.26	Eliminated
41.	5	10	0.33	Eliminated
42.	10	5	0.66	Appropriate
43.	11	4	0.73	Appropriate
44.	2	13	0.13	Eliminated
45.	15	0	1	Appropriate
46.	14	1	0.93	Appropriate
47.	3	12	0.2	Eliminated
48.	4	11	0.26	Eliminated
49.	14	1	0.93	Appropriate
50.	13	2	0.86	Appropriate
51.	10	5	0.66	Appropriate
52.	8	7	0.53	Appropriate
53.	2	13	0.13	Eliminated
54.	1	14	0.06	Eliminated
55.	14	1	0.93	Appropriate
56.	13	2	0.86	Appropriate
57.	15	0	1	Appropriate
58.	11	4	0.73	Appropriate
59.	12	3	0.8	Appropriate
60.	13	2	0.86	Appropriate

* CVR greater than 0.49, were accepted.

**Table-II:** Descriptive statistics of each subscale along with No. of items and internal consistency.

Subscales by Consensus (cSS): 1-5	Mean	Standard deviation	No. of items	Cronbach’s alpha
cSS1: Communication skills	3.77	0.06	7	0.405
cSS2: Self-Directed Learning	3.84	0.17	8	0.527
cSS3: Accountability	3.67	0.207	13	0.378
cSS4: Altruism	3.34	0.20	10	0.486
cSS5: Ethics	3.47	0.184	10	0.715
Overall scale	3.57	0.167	48	0.782

## DISCUSSION

The absence of a context specific, culturally sensitive and linguistically appropriate tool within the settings of Pakistani medical schools has inspired the need of development of a robust tool for assessment of professionalism. The study has shown encouraging results in assessing professionalism with high reliability scores for assessment of professionalism in the local Pakistani context. Reliability is an essential component of validity evidence. It is the degree to which the test yields the same result when repeated (reproducibility).^14^ Internal consistency of PAT was measured using Cronbach’s alpha. It reliably differentiates between the students who demonstrate the trait (i.e., professionalism) and those who do not. Different cut offs are used depending upon nature of examination. 0.90 for very high stakes exams, 0.80–0.89 is acceptable for moderate tests, 0.70–0.79 would be acceptable for lower stakes tests.^15^ The reliability of overall PAT calculated using Cronbach’s alpha was around 0.8 (0.783). Generally speaking, for affective measure reliability scores of 0.8 is considered as good score.^16^

Among the various tools cited in literature for assessment of professionalism, the most well-known valid, reliable and claimed as the first tool for assessment of professionalism in the medical profession was developed by Arnold et al.^17^ It was developed based on the ABIM Framework for Assessment of Professionalism. It measures professionalism as a comprehensive construct on the basis of operational definitions of professionalism by ABIM. The five domains identified in our tool were in accordance to American board of Internal Medicine (ABIM) framework of the international definition of professionalism which constitute six domains namely accountability, altruism, empathy, duty and excellence, respect, honesty and integrity.^18^ Most of the studies reported the assessment of professionalism in western context,^1^ there are very few studies pertaining to our sociocultural context.^18^ The uniqueness of PAT is that previous tools were constructed on basis of ABIM framework and mostly revalidated in western context.^17^ While in contrast PAT is constructed after extensive literature search and mutual consensus of experts using three Delphi rounds in local context. The tool developed by Arnold et al.^17^ was a 12-item scale, based on attributes of professionalism defined operationally by ABIM, with Cronbach’s alpha of 0.71 which is in comparison with our study which reported Cronbach’s alpha of 0.78.

The results of recent local qualitative study^18^ undertaken to explore the faculty perception regarding professionalism based on ABIM framework reported that all domains of ABIM framework could be utilized to define professionalism in Pakistani sociocultural and religious context. Study framed the domains of professionalism after the mutual consensus of the experts during Delphi rounds, the resultant factors were compared with the a priori factors (i.e., six elements of ABIM’s framework). In this study, the domains suggested by maximum experts during Delphi rounds were good interpersonal skills, being ethical and respectful. It has highlighted the importance of interpersonal skills while interacting with diverse team of medical fraternity.^18^ In accordance with our study, this study has also highlighted accountability as domain and its importance was discussed in Islamic context supporting the fact that Muslim medical students are self-accountable because of fear of Allah Almighty and Islamic teachings provide us the code of conduct and focused on being self-accountable in our deeds and actions.^18^ This is also in accordance with the study conducted in Arabian Context labelled as Arabian Learners’ Attitude of Medical Professionalism Scale (LAMPS),^19^ which also highlighted six domains as ABIM framework with addition of professional autonomy, which was therefore considered as the seventh element of professionalism in the Arabian context.^19^ Professional autonomy is due to cultural influence as in Arabian context, physician has more autonomy and power balance is more towards patients as compared to western world where there is concept of patient autonomy.^17^

LAMPS was developed and validated in the Arabian context. It has 28 items, five subcales and a reliability of 0.79.^20^ Cronbach’s alpha on the different subscales of LAMPS were as follows: on Subscale 1 “respect” with five items the alpha was 0.57, on Subscale 2 “autonomy” with six items it was 0.48, on Subscale-3 “Altruism” with 5 items was 0.42, on Subscale 4 Duty/Accountability it was 0.57, and Subscale-5 Honor/integrity had Cronbach’s alpha of 0.43. The Cronbach’s alpha obtained on the five Subscales of our study ranged from 0.40 to 0.78. Psychometric properties of the 28-item Persian version of Instrument of Professional Attitude for Student Nurses (IPASN) showed reliability of 0.89.^21^ In contrast to our study, Persian study reported that three out of six domains of ABIM construct can be used to define professionalism in Iranian context.^22^

### Limitations:

Data was collected from only one institute and only from one year (Year four MBBS) of undergraduate medical students. After running initial pilot test, it was reported by most of the faculty members that it’s not feasible to fill extensive 48 item tool, which warrants scale reduction using principal component analysis thus further establishing construct validity statistically to enhance feasibility and applicability of the tool. Moreover, we also aim to enhance generalizability of tool by testing its validity across different medical schools of Pakistan and at the regional level in order to enhance its cross cultural validity.

### Strengths

Despite all these limitations, the PAT can help to raise the bar of medical education in Pakistan and can be a way forward to enhance professionalism among medical students of Pakistan. This study can provide a strong foundation for teaching, learning and assessment of professionalism as part of formative and summative assessment.

## CONCLUSION

Professionalism assessment tool comprised of 48 items, across five subscales developed by mutual consensus and expert validation has high content validity and internal consistency. Thus it was established that professionalism assessment tool is reliable tool for assessment of professionalism in undergraduate medical students of Pakistan. The strength of this preliminary study focusing on development of tool is in its process of development and content validation by experts involving three rounds of Delphi.

### Recommendation:

To the best of our knowledge, this is the first tool developed for assessment of professionalism in the Pakistani context. It can be used as reliable tool for assessment of professionalism in undergraduate medical students. However, only content validity was established at this stage. Further study will be conducted to establish construct validity and subsequent scale reduction to enhance feasibility and applicability of the tool.

### Author`s Contribution:

**KN:** Contributed to the idea, questionnaire and data collection.

**RZ:** Contributed to the design, statistical analysis and drafting of the manuscript.

**KA & SF:** Contributed to data collection and critical review.

All authors take equal responsibility for the accuracy and integrity of the work.

## APPENDIX

### Professionalism Assessment Tool (PAT) for assessment of professionalism in undergraduate medical student

**Table:**

Student Roll No.	Year MBBS	1	2		3	4	5
Evaluator Name	Department	Basic Sciences Faculty		Clinical Faculty		Community Health Sciences Faculty	

**INSTRUCTIONS**

- Carefully read each item statement before marking the response.
- Use the scale: not observed, seldom, once in a while, sometimes, usually, often, always
- For each of the numbered statements given below, please give your response to the posed items by marking [x] in the appropriate box. This response is on an 8-item scale from 0-6.

***Please use each number only once***.

### Interpretation of descriptors:

**Table:**

*Not observed*	*Seldom*	*Once in a while*	*Sometimes*	*Usually*			*Often*	*Always*		*U/C*			
0	1	2	3	4			5	6		Unable to comment			
				0			1	2	3	4	5	6	U/C
1.Effective Communication skills:													
i) Communicate effectively with patients and their families													
ii) Show respect to peers, physicians and other health professionals													
iii) Use collaborative negotiation to resolve conflict, anger, confusion and misunderstanding													
iv) Address challenging communication issues effectively, including informed consent, breaking bad news													
v) Communicate effectively in different cultural contexts													
vi) Avoids offensive speech & unfair criticisms to others													
vii) Communicate effectively using listening, verbal, non-verbal, questioning, explanatory and writing skills													
Maximum score													42
2.Commitment to Competence: (Self-Directed Learning)					0	1		2	3	4	5	6	U/C
i) Keep knowledge and skills up to date													
ii) Review and reflect on his/her own performance													
iii) Responds positively to constructive criticism													
iv) Seek and endorse diverse perspectives of team members to foster creative problem-solving													
v) Seeks self-improvement													
vi) Strives to meet quality standards as represented by appropriate benchmarks													
vii) Show leadership skills and initiative													
viii) Participates in activities aimed at attaining excellence in medical education & patient care													
Maximum score													48
3.Duty & excellence:					0	1		2	3	4	5	6	U/C
i) Promote patient safety													
ii) Demonstrate awareness of own limitation													
iii) Admits error and omission													
iv) Demonstrate effective time management /punctuality													
v) Completes task in reliable manner													
vi) Is cognizant of own gaps in knowledge & skills													
vii) Avoids deprecating language													
viii) Understands risk benefit & cost effectiveness													
ix) Aware of disparity of health needs of society													
x) Accountable to patients, profession& society													
xi) Shows compassion towards patient													
xii) Shows empathy towards patient													
xiii) Conforming to social norm													
Maximum score													78
					0	1		2	3	4	5	6	U/C
4.Altruism :													
i) Maintain professional relationship with patient													
ii) Respect patient’s autonomy													
iii) Do not exploit patients’ privacy & financial incentives													
iv) Demonstrate responsiveness to patient needs that supercede self-interest													
v) Support colleague’s professional development													
vi) Volunteer services focusing on improving the overall health and well- being society													
vii) Encourage proper distribution of health care resources													
viii) Actively contribute towards institutional goals													
ix) Adopts uniform and equitable standards													
x) Understand community needs & bias towards vulnerable populations													
Maximum score													60
5.Ethics:					0	1		2	3	4	5	6	U/C
i) Maintain patient confidentiality													
ii) Maintains respectful relationship with patient													
iii) Reports medical or research errors													
iv) Discloses conflicts of interest in the course of professional duties and research activities													
v) Demonstrate sensitivity and responsiveness to patients’ culture, age, gender, and disabilities													
vi) Maintains of anonymity of patient records													
vii) Meets commitments &obligations in conscientious manner													
viii) Reports data consistently, accurately and honestly													
ix) Commitment to honesty with patients													
x) Maintain personal and professional code of conduct													
Maximum score													60
**Global Rating: 0-6**													
**Total Numbers = 288**													
*Unacceptable*		*Needs improvement*		*Borderline*		*Meets Expectations*		*Satisfactory*		*Good*		*Excellent*	
0		1		2		3		4		5		6	
